# Co-Designing Culturally Grounded Mobile Health Games for Hypertension Management in Indigenous Communities

**DOI:** 10.1145/3663547.3746321

**Published:** 2025-10-22

**Authors:** Alison Graham, Tochukwu Arinze Ikwunne, Jared Duval

**Affiliations:** School of Informatics, Computing, and Cyber Systems, Northern Arizona University, Flagstaff, Arizona, USA; Informatics, Computing, and Cyber Systems, Northern Arizona University, Flagstaff, Arizona, USA; Informatics, Computing, and Cyber Systems, Northern Arizona University, Flagstaff, Arizona, USA

**Keywords:** Hypertension Management, Game and Play, Indigenous Health, Digital Health Interventions

## Abstract

Hypertension, which disproportionately affects older adults, continues to pose a critical health challenge among Indigenous communities, where systemic healthcare inequities and cultural mismatches in medical interventions contribute to high rates of undiagnosed and unmanaged cases. This paper explores the co-design of culturally relevant mobile health games to support hypertension management among Indigenous Peoples in Northern Arizona. Drawing from participatory design methodologies, we conducted a workshop with 14 participants to conceptualize and prototype mobile applications focused on five key factors of hypertension care: medication adherence, nutrition, exercise, education, and blood pressure monitoring. The workshops revealed that culturally rooted play and storytelling may significantly enhance user engagement and acceptance of digital health tools. Using insights from these sessions, we developed five medium-fidelity prototypes that reflect diverse game and play strategies, from traditional gamification techniques to reappropriations of existing games infused with Indigenous cultural elements. Our findings highlight how participatory and culturally grounded design approaches can produce mobile health interventions that may resonate more deeply with Indigenous worldviews and healthcare practices. This study contributes empirical design artifacts and knowledge to the field of accessible health technologies, emphasizing the importance of Indigenous-led co-design in addressing chronic disease disparities. We conclude by outlining future directions for iterative prototyping and playtesting, with implications for broader applications in culturally responsive digital health innovation.

## 1 Introduction

Hypertension remains a pressing health issue among older Indigenous communities, exacerbated by systemic disparities in healthcare access, cultural barriers, and the lack of tailored digital health interventions [3]. Several factors contribute to these disparities, including the lack of health education materials in Indigenous languages, travel distances to healthcare providers, limited awareness, skepticism toward Western healthcare, and the isolation of rural communities [4]. Additionally, many Indigenous communities are classified as food deserts, where access to fresh food is limited, furthering the risk of hypertension [1]. Socioeconomic challenges, including limited access to insurance, further compound these barriers, leading to lower rates of diagnosis, treatment adherence, and overall hypertension management [4]. The challenges that motivate this research meet all 10 characteristics of a *Wicked Problem* and are thus well-suited to a *Research through Design* (RtD) approach, which generates complexity, situated knowledge and understanding through the iterative creation and reflection on design artifacts [5]. Hypertension disproportionately affects Indigenous populations, with studies showing prevalence rates significantly higher than in non-Indigenous groups. These disparities contribute to an increased risk of cardiovascular disease, kidney failure, and other complications [28–30]. The high burden of hypertension in Indigenous communities underscores the need for interventions tailored to their socio-cultural and geographic contexts.

In a preliminary synthesis of prior literature, we identified five factors that are critical for effective hypertension management: medication adherence, education, exercise, nutrition, and blood pressure monitoring. Medication adherence is often low among Indigenous populations due to distrust of Western medicine, cost barriers, and side effects [3]. Providing culturally relevant education about risks and treatment options can improve awareness and self-efficacy. Exercise, particularly traditional physical activities, can be integrated into game-based interventions to encourage movement and cardiovascular health. Nutrition remains a challenge due to food insecurity and reliance on processed foods, necessitating tailored dietary guidance [3, 6]. Finally, consistent blood pressure monitoring is essential for disease management, yet many Indigenous individuals lack access to home monitoring tools or regular clinical visits [7]. Telehealth games may address these challenges, as evidenced by historical examples that offer motivating contexts that lead to sustained interventions accompanied by improved health outcomes (e.g., in speech therapy games [8, 9], a tobacco cessation game for young people [10], virtual reality stroke rehabilitation games [11, 12]), culturally appropriate health education (e.g., a behavioral therapy game for Māori youth [13]), and gamified tracking tools that align with Indigenous rural lifestyles [14] (e.g., Duval’s internet equity game [15]). With the insight that games have the potential to offer culturally relevant and sustained interventions for improving health outcomes among marginalized populations, the following research questions drove this work:

***RQ1*:** How do the design particulars and cultural representations embedded in co-created mobile health game prototypes reflect Indigenous epistemologies?
***RQ2*:** What forms of intermediate design knowledge and design patterns emerge from co-designing culturally grounded mobile health games for hypertension management?

The methodologies employed in this study include a workshop grounded in participatory design [16] and *Research through Design* [20] principles, medium-fidelity prototyping [17], and the construction of an annotated portfolio [18] to surface intermediate design knowledge [19] across the resulting artifacts. We conducted a co-design workshop with 14 Indigenous community members to explore culturally relevant, playful approaches to hypertension management. These sessions emphasized collective storytelling, culturally grounded play, and participants’ lived experiences, leading to the conceptualization of five mobile game prototypes. Each prototype addresses a different facet of hypertension care, resulting in unique design particulars shaped by participant input. Following the workshops, we developed medium-fidelity interactive prototypes that embodied the co-created ideas in concrete, testable forms for future research. We constructed an annotated portfolio [18] to analyze and communicate the design insights generated through this process. This portfolio enabled us to compare the prototypes side-by-side, identify recurring design patterns and culturally rooted design logic, and reflect on Indigenous epistemologies in participatory design. Through this approach, we foregrounded the artifacts, and the broader cultural, methodological, and experiential knowledge embedded within them. This *Research through Design* [20, 21] study makes two key contributions to the field of accessible and culturally responsive playful health technologies for older Indigenous adults:

### Artifact:

Five medium-fidelity mobile game prototypes co-designed with Indigenous community members, each addressing a key dimension of hypertension care via culturally grounded play and storytelling.

### Intermediate Design Knowledge:

An annotated portfolio that bridges design particulars with common design strategies, including gamification, reappropriation, integration of cultural narratives, incentivized progression, and playification to create culturally responsive playful health technologies.

We report multiple findings related to design particulars embedded in the co-created games that reflect Indigenous epistemologies (RQ1) and to the forms of intermediate design knowledge [19] that emerge from the co-design process (RQ2). Related to design particulars and Indigenous epistemologies, our findings show, for example, that culturally grounded play and storytelling expressed through familiar formats like Jeopardy with traditional narratives about animal care and communal movement practices can enhance the cultural resonance of mobile health interventions. The five co-designed prototypes address core aspects of hypertension care, each uniquely shaped by the values, experiences, and knowledge systems shared by Indigenous participants. Related to the forms of intermediate design knowledge [19] that emerge from the annotated portfolio [18], our findings show, for example, that common design patterns in HCI are reflected in and resonate with participatory design workshops with Indigenous stakeholders, including Gamification [22], the reappropriation of familiar game formats [23], Situated Play Design [24] that integrates cultural narratives [25], incentivized progression [26], and Playification [27]. These patterns demonstrate how co-design processes can generate transferable strategies that inform the development of playful health technologies for hypertension management in Indigenous communities.

The remainder of this paper is structured as follows: we begin by reviewing related work on culturally grounded health interventions, telehealth, and game-based health technologies, followed by a discussion of methodological frameworks that support participatory and culturally responsive design (Section 2). We then describe our study protocol, including the co-design workshop, prototype development, and the construction of an annotated portfolio (Section 3). This is followed by a presentation of the annotated portfolio (Section 4). We conclude with a discussion of the broader implications for designing playful health technologies for Indigenous communities as they relate to both research questions and offer recommendations for future research and culturally attuned mobile health development (Section 5).

## 2 Background

This section begins by providing related works that motivate the need for culturally relevant hypertension management solutions for Indigenous communities, emphasizing the systemic and structural barriers that contribute to health disparities (Section 2.1). Next, we provide inspiring works related to the role of telehealth and game-based interventions in enhancing hypertension management for Indigenous populations (Section 2.2). Finally, we explore the short-comings of conventional digital health interventions and provide methodological considerations to facilitate participatory design workshops that are both culturally relevant and technologically effective (Section 2.3).

### 2.1 Prevalence of Hypertension among Indigenous Communities

Hypertension disproportionately affects Indigenous populations, with studies showing prevalence rates significantly higher than in non-Indigenous groups [30]. Research indicates that Indigenous adults in the U.S. experience hypertension at rates ranging from 30% to 40%, often coupled with lower diagnosis and treatment rates [28, 29]. Indigenous individuals often also face challenges in accessing primary care services, leading to delayed diagnoses and inadequate management [31]. Historical and ongoing mistrust of Western healthcare systems further complicates treatment adherence, resulting in poorer health outcomes [32]. Socioeconomic factors such as limited health insurance coverage and high poverty rates further hinder access to healthcare services, medications, and preventive care [33]. Furthermore, many Indigenous communities reside in food deserts, where fresh food is scarce, leading to diets high in processed and sodium-rich foods that contribute to elevated blood pressure levels [34]. These structural and environmental challenges make hypertension a pressing public health concern, requiring targeted and culturally responsive interventions.

### 2.2 The Role of Telehealth and Game-Based Interventions

Recent studies support the use of telehealth and play-based interventions as effective tools for managing chronic conditions, particularly in underserved populations [38]. Inspiring examples include play-based interventions for motor and social skills of children with Autism Spectrum Disorder [39], a telehealth program for children with Prader-Willi Syndrome [40], and a community-based play group to prevent developmental delays in rural settings [41]. Telehealth platforms increase accessibility to resources by providing monitoring and educational tools to improve patient self-management, bridging gaps in healthcare accessibility for rural communities [42]. Additionally, games provide an engaging medium for health education and behavioral change, allowing users to develop self-management skills in an interactive environment [43]. Game-based health interventions have been shown to improve engagement [44], motivation [45], and adherence to treatment plans [46]. By combining these two approaches, telehealth games offer an innovative strategy to bridge healthcare gaps, making hypertension management more accessible and engaging for Indigenous communities.

### 2.3 Participatory Design for Indigenous Health Technologies

Addressing hypertension in Indigenous communities requires an approach that acknowledges and integrates traditional knowledge systems, culturally appropriate health education, and accessible digital health interventions [35]. Most health applications are designed for a broad audience, neglecting the lived realities and cultural, linguistic, and socioeconomic factors that influence engagement and adherence in Indigenous communities [36, 37]. Research underscores the importance of tailoring health interventions to align with Indigenous knowledge systems, traditional practices, and holistic approaches to well-being [37]. By incorporating Indigenous perspectives, healthcare providers and researchers can develop more effective strategies to improve hypertension outcomes while fostering trust and accessibility within Indigenous communities.

Human-Computer Interaction research emphasizes the importance of participatory design in creating accessible and inclusive technologies [16]. Participatory design engages end users in the development process, ensuring that their needs, preferences, and lived experiences inform design decisions [46]. Research through Design [20, 21] offers a powerful methodological approach for integrating Indigenous ways of knowing with digital health interventions to improve hypertension outcomes. Unlike traditional research methods, Research through Design emphasizes an iterative, exploratory, and co-creative process that facilitates the inclusion of culturally meaningful engagement strategies, such as gamification rooted in traditional activities [47]. By engaging Indigenous community members in designing and developing health technologies, Research through Design fosters mutual learning and ensures that digital solutions reflect cultural values, lived experiences, and traditional health practices [48]. This participatory approach has the potential to enhance the usability and acceptance of health interventions, strengthen trust in digital healthcare solutions, and address longstanding skepticism toward Western medical models [32, 37].

## 3 Methodology

This study employed a participatory research approach to co-design and evaluate culturally relevant mobile health games for hypertension management in Indigenous communities. We begin by providing details about ethics (Section 3.1), recruitment (Section 3.2), and participant demographics (Section 3.3). Finally, we provide an overview of our protocol and analysis. The research protocol was structured into three key phases: workshopping (Section 3.4), medium-fidelity prototyping (Section 3.5), and developing an annotated portfolio (Section 3.6).

### 3.1 Ethics

Given the sensitive and community-centered nature of the work, we followed established protocols for informed consent and received IRB approval, which included approved tribal consultation. Participants were informed of the study’s purpose, their right to withdraw without consequence, and how their contributions would be used. In line with Indigenous data sovereignty principles, we emphasized participant confidentiality, transparency, and non-extractive knowledge sharing throughout the process.

### 3.2 Recruitment

Participants were recruited through community networks, educational institutions, and Indigenous academic partners. Recruitment materials emphasized the collaborative nature of the research and the goal of co-creating culturally relevant mobile health games for hypertension management. Recruitment efforts prioritized respectful engagement and transparent communication, recognizing the history of extractive research practices in many Indigenous communities.

### 3.3 Participant Demographics

A total of 14 Indigenous participants took part in the co-design workshop. The participants reflected diverse indigenous cultural and geographic contexts. Participants self-identified as members of a range of Indigenous Nations and communities with 1 participant from the following communities: Hopi, Navajo, Diné, Chickasaw, Hidatsa, Oneida, and native Hawaiian. There were 7 participants who did not disclose their affiliation. Ages ranged from 29 to 60 years old, and all participants were female.

### 3.4 Workshopping

**Figure 1:**
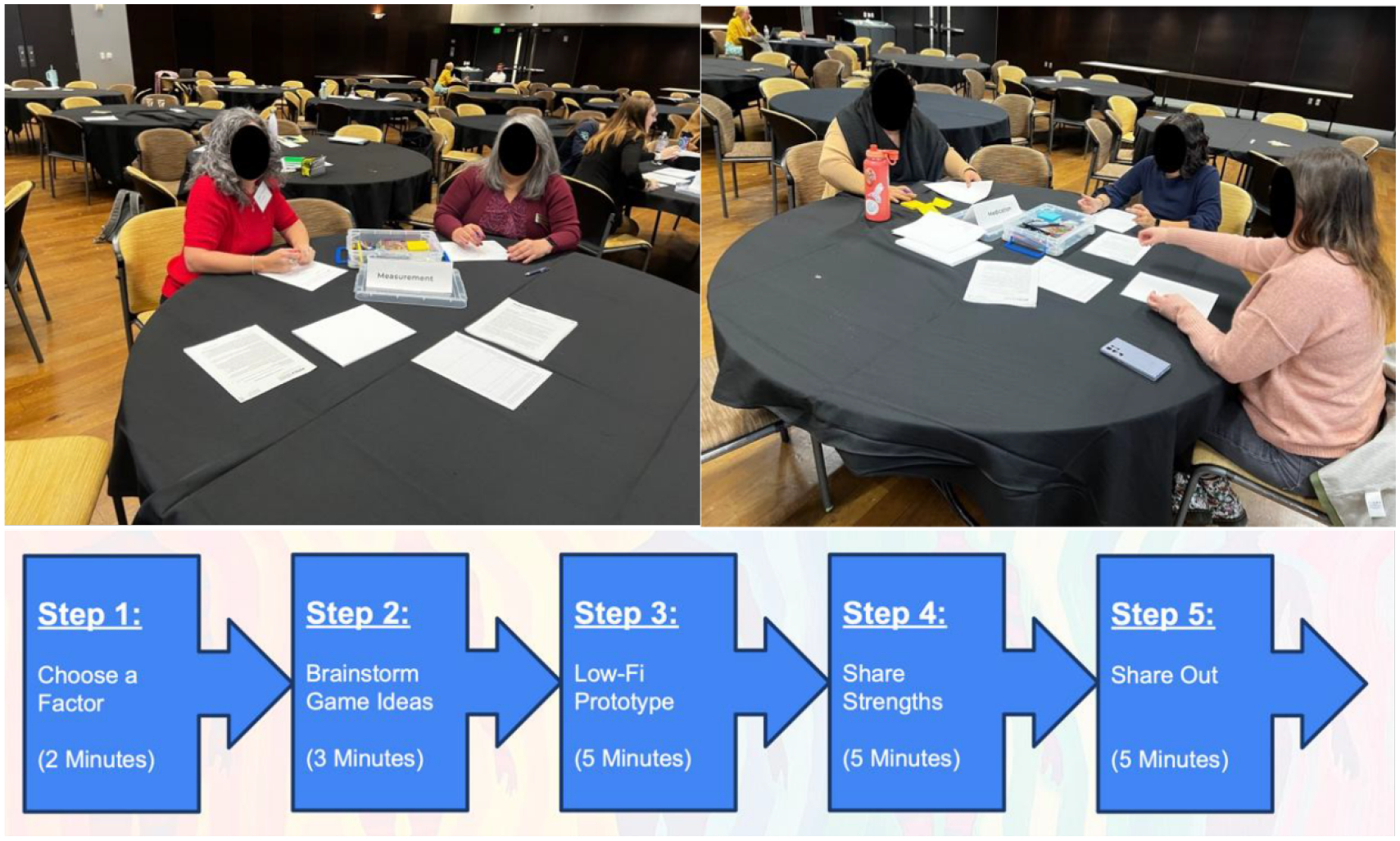
On the top left, two participants work on their prototypes in the measurement group. On the top right, three participants work on their prototypes in the medication group. On the bottom, a line of boxes and arrows denote the series of steps taken during the workshop.

To initiate the co-design process, we conducted a 2-hour participatory design workshop with Indigenous community members. The context of the workshop was within a conference on the topic of *Indigenous Computational Futures*. The workshop aimed to discuss hypertension management needs, brainstorm culturally relevant design elements, and co-create concepts for mobile health games. Participants had time to discuss hypertension, brainstorm ideas, and reflect before beginning the co-design process. The co-design section of the workshop was split into 5 steps; choose a factor, brainstorm, sketch low-fi prototype, share strengths, and share out (Figure 1). The participants split themselves into 5 groups and were encouraged to explore the idea of play and create game-based solutions that align with Indigenous ways of knowing and cultural practices. Participants then worked through the steps as a group before all the participants regrouped to share out their ideas.

### 3.5 Medium-fidelity prototyping

Based on the insights gathered during the workshops, we developed medium-fidelity prototypes. These prototypes incorporated key ideas identified by participants, such as culturally relevant themes, interactive storytelling elements, and game and play strategies that encourage adherence to the five factors for effective hypertension management. We started by creating 17 app designs based on the workshop outputs and sticky note feedback. We synthesized over-lapping features through affinity diagramming to create one app for each management factor. Many of the gamification elements, including progress tracking, point systems, and narrative-driven tasks, were proposed by participants during the workshop. Additional mechanics were introduced by the research team using design patterns informed by Indigenous play and models of culturally responsive health behavior change. The prototypes were created using Figma to provide interactive wireframes and preliminary gameplay mechanics that could be tested with users.

### 3.6 Annotated Portfolio

To synthesize insights across the five medium-fidelity prototypes, we constructed an annotated portfolio [18] as a means of articulating intermediate design knowledge. This process involved systematically analyzing each prototype in relation to its design goals, cultural grounding, gameplay mechanics, and participant-derived concepts. We began by documenting the key design particulars of each game, such as the use of familiar formats (e.g., Jeopardy), integration of traditional narratives (e.g., animal care practices), or specific mechanics (e.g., rewards for blood pressure measurement.) Through iterative comparison, we inductively identified common strategies that spanned multiple prototypes, including gamification, reappropriation, playification, incentivized progression, and the integration of cultural narratives. These recurring patterns were organized and annotated in relation to the specific cultural and participatory contexts from which they emerged. The annotated portfolio enabled reflection on how each prototype embodied Indigenous epistemologies and supported the abstraction of design patterns that may inform the future development of culturally responsive, playful health technologies.

## 4 Annotated Portfolio

Building on the participatory workshops and subsequent medium-fidelity prototyping, the annotated portfolio serves as a structured means to analyze and reflect on the points that emerged through the co-design process. Each prototype is examined its design particulars, cultural grounding, creation process, and the feedback received during prototyping. This section highlights the co-design process and how it relates to the development of culturally responsive, engaging, and accessible health technologies.

### 4.1 Hypertension Education Game

**Figure 2:**
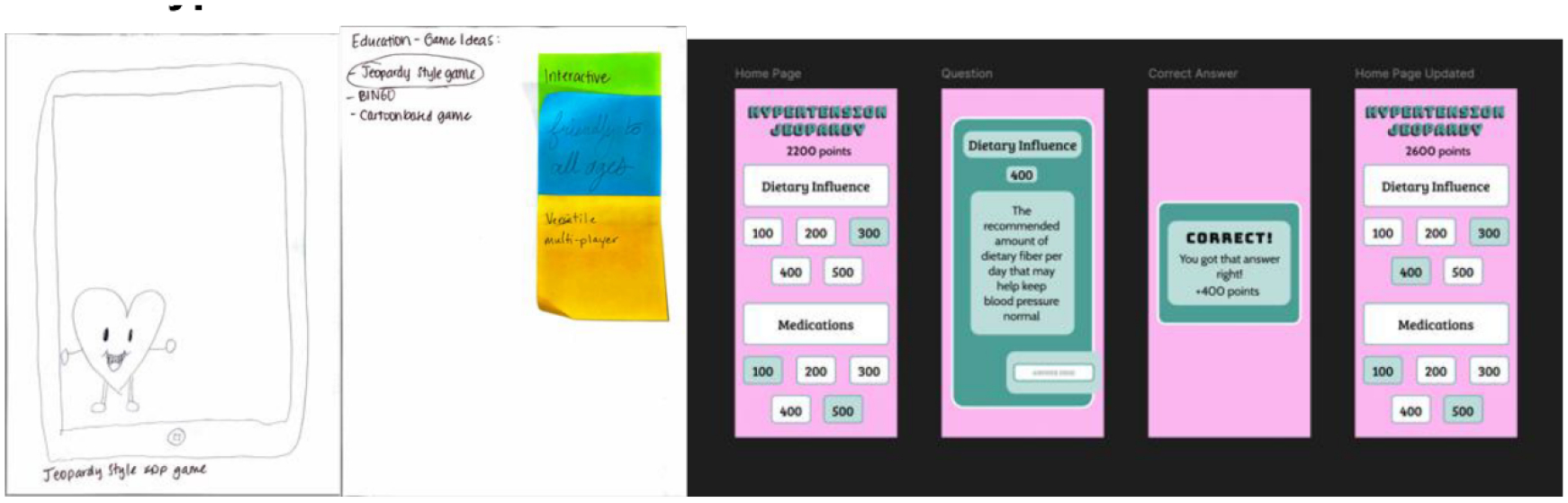
On the left, the low-fidelity paper prototype. In the center, positive aspects of the design were assigned by peers using Sticky Notes. On the right is the resulting medium-fidelity prototype.

The Hypertension Education Game (Figure 2) draws upon game-based learning principles to engage users in self-management of hypertension through knowledge reinforcement. The translation from low- to medium-fidelity for this game began with identifying the core mechanic of the participant prototype: a Jeopardy-style trivia board. We maintained the point-grid system and recreated it ensuring that each selection led to a corresponding question-and-answer flow. The category-based layout, reminiscent of familiar game show structures, is meant to reduce cognitive load and make health education feel approachable and fun. Multiple design revisions were made to fine-tune contrast, layout, and the messaging to ensure the app felt both playful and informative without becoming overly gamified or overwhelming. The use of point values and positive feedback messages are intended to support both extrinsic motivation and cognitive engagement. The feedback, in the form of sticky notes, from the workshop captured participants’ enthusiasm for this app’s structure and tone. Comments like “interactive,” “friendly for all ages,” and “versatile” highlight how the format was perceived as inviting and accessible. The idea of using a quiz format with selectable categories was originally proposed by participants as a way to make health knowledge feel rewarding and communal. These reflections support the app’s potential to encourage group-based play, making hypertension education a shared activity rather than an isolating one.

### 4.2 Hypertension Diet Game

**Figure 3:**
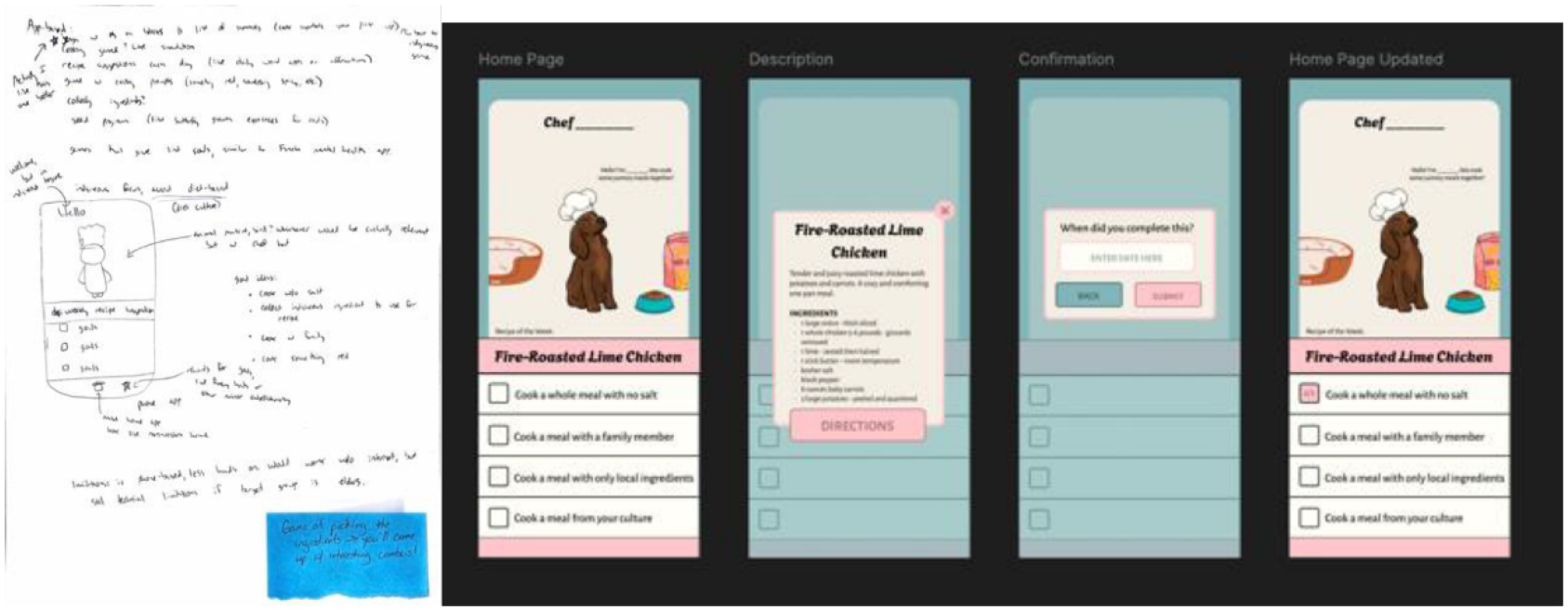
On the left, the low-fidelity paper prototype with a Sticky Note attached, which compliments the design of the game. On the right is the resulting medium-fidelity prototype.

The Hypertension Diet game (Figure 3) uses character-driven motivation, clear goal-setting, and culturally-inclusive task options to encourage users to manage their diet. The original prototype for this game was the most developed of the low-fi prototypes, making it the easiest to translate. In the digital version, we developed a welcoming onboarding screen featuring an animal named “Chef ____,” accompanied by a list of goal-based cooking tasks. We created simple progress-tracking mechanics that mirrored the checklist-style logic from the workshop sketches. The inclusion of a date entry screen, where users could log completion, helped reinforce the task- and time-based tracking seen in the original prototype. Each of these core mechanics—character-driven motivation, checklist-style goals, and food preparation as progress—was introduced by participants during the workshop and expanded by the research team during digital prototyping. Additional refinements, such as the friendly onboarding flow and progress animations, were designed to align with Indigenous models of supportive guidance and everyday celebration. Across all revisions, we prioritized making the goals feel achievable and supportive rather than prescriptive, echoing the co-design emphasis on cultural sensitivity and non-judgmental health support. For this game, a participant noted, “game of picking ingredients → you’ll come up w/ interesting combos!”—highlighting the potential to extend the current recipe-based design into a more interactive, playful experience. Such insights from co-design participants reflect meaningful user engagement with the prototypes and offer actionable directions for iterative development.

### 4.3 Medication Adherence Game

**Figure 4:**
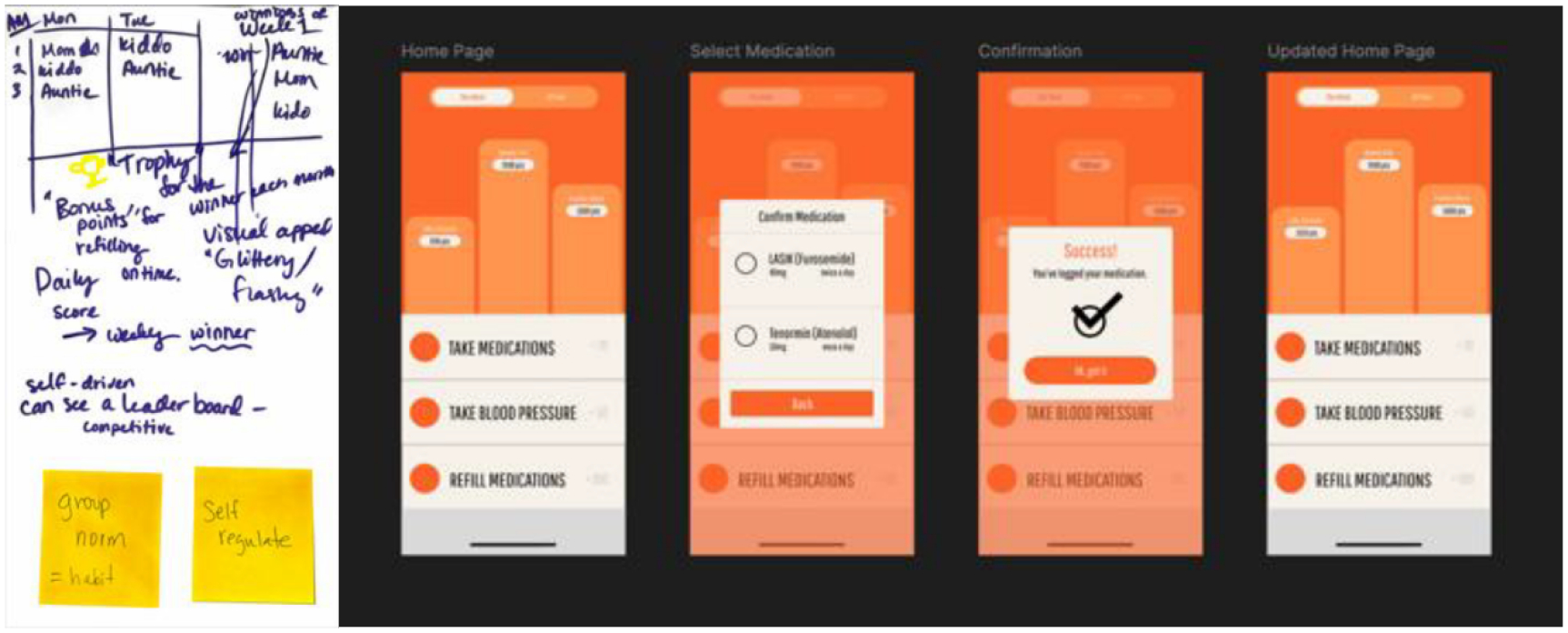
On the left, the low-fidelity paper prototype with a Sticky Notes attached, which compliments habit building features. On the right is the resulting medium-fidelity prototype.

The Medication Adherence Game (Figure 4) uses a leaderboard and points system to offer immediate positive feedback for adherence behaviors such as taking medication and refilling prescriptions. The participants’ low-fidelity design for the Medication Adherence Game emphasized visual feedback and routine reinforcement, specifically through a point-based system and leaderboard. To preserve this behavioral framing, the medium-fidelity prototype featured a simplified user journey: logging medication, viewing progress, and seeing one’s placement on a leaderboard. Confirmatory messages like checkmarks and “Success!” were added after each log, to visually reinforce routine formation. The idea of using social visibility to encourage habit formation was introduced during the workshop, where participants described how collective tracking could reduce stigma and create mutual accountability. For this game, sticky note comments included “group norm = habit” and “self-regulate,” suggesting that participants viewed the leaderboard and gamified routine as mechanisms for fostering habitual behavior through social comparison and accountability. These reflections support the idea that visualizing adherence among peers may reinforce motivations, potentially contributing to sustained self-management behaviors.

### 4.4 Blood Pressure Measurement Game

**Figure 5:**
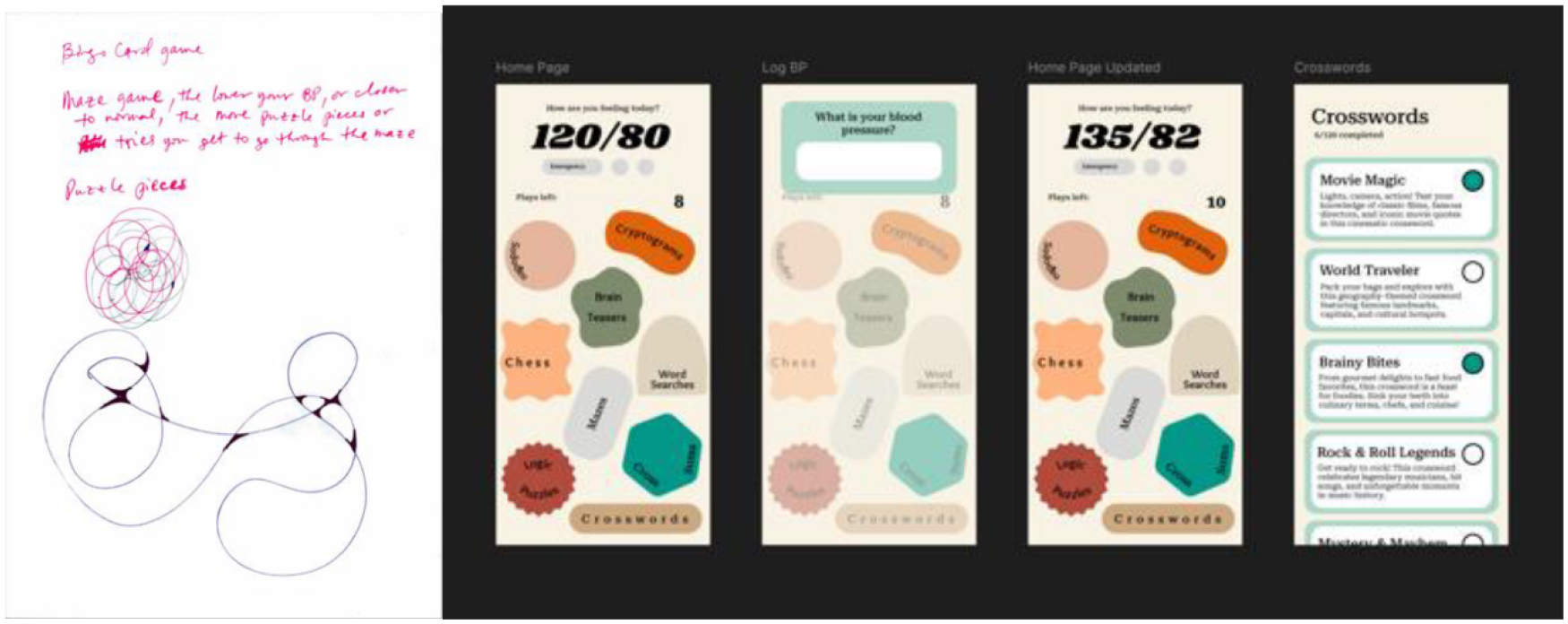
On the left, the low-fidelity paper prototype. On the right is the resulting medium-fidelity prototype.

The Blood Pressure Management Game (Figure 5) uses playful and organic visual elements, such as irregularly shaped buttons and soft color palettes, selected to create a non-clinical, approachable experience. For the transition from low- to medium-fidelity, this game had very little details in its sketch, leaving much of this app’s design and functionality open ended. The original participant sketches combined blood pressure logging with cognitive mini-games. To reflect this in the digital prototype, we first developed a clear input screen for entering and displaying current blood pressure data at the top of the home screen. Below this, we introduced organically shaped buttons for game types like “Sudoku,” “Word Search,” and “Crosswords,” pulling visual inspiration from mobile game interfaces to make the experience inviting and non-clinical. The concept of pairing measurement with leisure activities was present in participant sketches, where cognitive games were positioned as a way to reduce anxiety around health data. The integration of self-monitoring (e.g., blood pressure input and display) alongside cognitively engaging activities facilitates a seamless transition between health tracking and leisure-based interventions. This game did not receive any feedback notes.

### 4.5 Exercise Game

**Figure 6:**
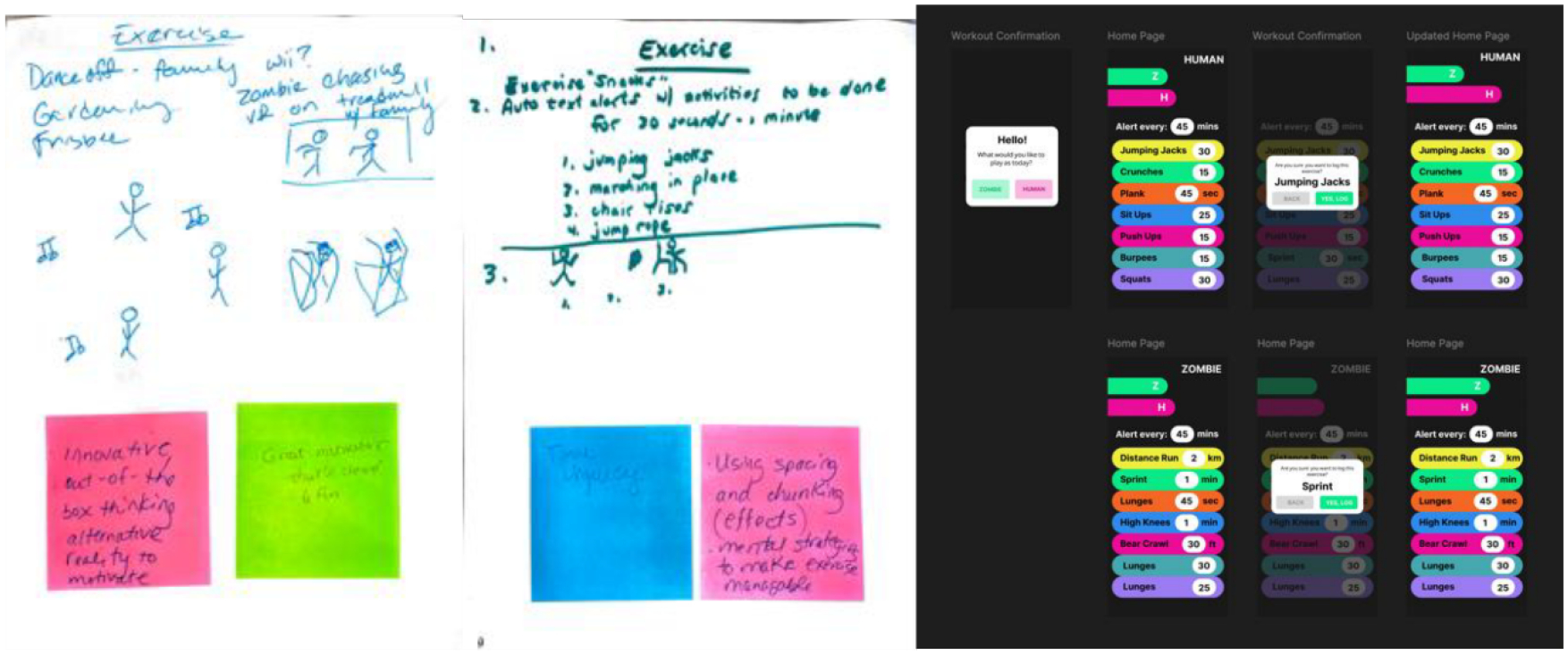
On the left, the low-fidelity paper prototypes with Sticky Notes attached, which compliments the creative design of the game. On the right is the resulting medium-fidelity prototype.

The Exercise Game (Figure 6) presents a playful, gamified physical activity tracker where users choose to play as either a “HUMAN” or a “ZOMBIE,” each with its own list of exercises. This games original prototype featured a playful mechanic, where users could use virtual reality to simulate being chased by zombies. Translating this into a functional prototype required preserving the creative spirit of the design while altering the activity prompts. We began by designing an avatar selection screen with large, vivid buttons and creating two roles to further play capabilities. Each avatar linked to a corresponding exercise list with clear activity labels and durations. We added a recurring timer element at the top of the screen, which aligned with participants’ time-based structure and sense of urgency noted. The idea of role-playing through themed characters like zombies and humans came directly from participants’ sketches, which emphasized fun and narrative as motivators for physical movement. Co-design participants responded positively to the app’s imaginative framework. Sticky notes described it as showing “innovative out-of-box thinking,” “alternative reality to motivate,” and “great motivator.” These comments suggest that the app’s narrative framing successfully captured users’ interest while maintaining a focus on real-world health behaviors. The blend of creativity and functionality resonates with the co-design values of making health interventions both culturally resonant and personally meaningful.

## 5 Discussion

Drawing from the insights generated through the co-design process and annotated portfolio, we consider how the design particulars of the game prototypes reflect Indigenous epistemologies and lived experiences. We also examine the emergence of intermediate design knowledge and its potential to inform future work in culturally grounded digital health interventions. This discussion situates our contributions within the larger discourse on participatory design, health equity, and playful health technologies, and offers recommendations for advancing culturally attuned mobile health development for Indigenous communities.

### 5.1 Design Particulars and Indigenous Epistemologies (*RQ1*)

#### 5.1.1 Education Game:

The education game—framed as a Jeopardy-style trivia game—affords players the opportunity to construct, navigate, and reframe categories of knowledge in ways that reflect epistemic self-determination. As Jeopardy prompts are reinterpreted or co-created by the player, the game allows for the emergence of personally or culturally salient domains of knowledge rather than privileging a universalized or biomedical hierarchy. This aligns with McCarty’s framing of Indigenous education as a process of “self-determining knowledge construction” that resists imposed curricular norms in favor of localized, meaningful knowings [49]. In this way, the module does not position knowledge as fixed content to be absorbed, but rather as a generative domain for co-creation and reinterpretation.

#### 5.1.2 Diet Game:

The diet module invites players to make food choices guided by a companion character—often an animal like a dog—who embodies warmth, encouragement, and accountability. This design aligns with Indigenous relational epistemologies in which animals are not metaphorical, but co-participants in knowledge-making. The act of cooking ”for” or ”with” an animal character echoes practices of care and reciprocity with nonhuman kin, reinforcing ethical obligations embedded within everyday acts like meal preparation. Through this mechanic, the game surfaces a more-than-human perspective that reframes food as not merely functional, but relational [50].

#### 5.1.3 Medication Game:

The medication game centers on collective success via shared progress tracking and group incentives—an intentional departure from individualistic health logging. This resonates with Indigenous epistemologies of distributed cognition and “group mind,” in which knowledge, decision-making, and well-being are shared responsibilities of the collective rather than isolated individuals. This collective framing echoes “whānau decision-making,” a process in which group cohesion shapes health behavior and meaning-making. Within the game, this is operationalized via communal leaderboards and group achievements, embedding shared responsibility into the act of adherence [51].

#### 5.1.4 Blood Pressure Measurement Game:

The blood pressure module subtly frames biometric self-monitoring as a gateway to pleasure and relaxation, a dynamic that aligns with Indigenous temporalities that link work and reward through rhythm and responsibility [52]. The act of measuring blood pressure is scaffolded not as a chore, but as part of a cycle that permits subsequent joyful or leisure activity. This sequencing reflects an Indigenous ethic of balance between contribution and enjoyment, where pleasure is earned through meaningful participation and care for the self and community [53].

#### 5.1.5 Exercise Game:

The exercise module is designed around loose prompts for movement, enabling playful improvisation and story-making through the body. These features resonate strongly with Indigenous traditions of storytelling as embodied knowledge, where narrative is not bound to text but enacted through song, dance, and motion [54]. Moreover, the game supports co-located or parallel play, inviting intergenerational and communal participation [55]. This aligns with Indigenous practices of communal movement—ceremonial, functional, or recreational—that bind people together across time and space through collective action [54]. The game thus extends movement from a physical necessity to a narrative act of becoming-with others.

### 5.2 Intermediate Design Knowledge (*RQ2*)

#### 5.2.1 Education Game:

The design of the education game encourages reappropriation by offering ambiguous play prompts and loosely structured activities, drawing on the concept of ambiguity as a resource for design [56]. Much like the cultural probes approach [57], this module invites participants to explore cognitive challenges (e.g., logic puzzles, crosswords) on their own terms, leaving room for personalization. This openness resonates with work on Spellcaster [58, 59], which demonstrated how users adapt therapeutic games to meet their own emotional needs. By not enforcing specific cognitive tasks, the education game affords players the opportunity to reframe the experience—potentially as leisure, routine, or even competition. These dynamics reflect patterns from our systematic review, where “Totally Fun First” games often succeed by enabling players to shape the game experience to fit their daily rhythms and affective contexts.

#### 5.2.2 Diet Game:

The diet game design aligns closely with Situated Play Design (SPD) [60], in which play is embedded within everyday activities and personal meaning-making. Drawing inspiration from Human-Food Interaction Research [61], the game invites users to engage with cooking tasks that intersect with their identities, such as preparing a meal from their culture or cooking with family members. This supports cultural narrative engagement, whereby users recontextualize food preparation as both a nutritional and relational act. The game’s light roleplay elements (e.g., the dog chef guide) mirror “Animals for Self-Care” [62], leveraging character-led framing to scaffold self-reflection and emotional warmth. Furthermore, like cultural probes, the module provides structure while maintaining interpretive openness, encouraging players to integrate their own culinary practices and familial traditions into the game.

#### 5.2.3 Medication Game:

The medication adherence module explicitly draws from gamification techniques, incorporating points, leaderboards, and rewards—hallmarks of The Gameful World [22]. This design scaffolds shallow engagement through recognizable tropes (e.g., +10 for taking medications), but offers room to deepen through social comparison and identity formation. Although effective at providing extrinsic motivation, this approach risks instrumentalization. Playification would suggest further enhancing this module with more meaningful player-driven mechanics—transforming routine logging into a playful and expressive interaction [63]. The current design stands at the threshold between gamification and playification, offering a potential trajectory for refinement through deeper, context-aware mechanics.

#### 5.2.4 Blood Pressure Measurement Game:

This module employs incentivized progression by integrating clear, measurable rewards for checking and logging blood pressure. The psychological mechanisms at play here mirror classic reward and incentive structures, leveraging timing, points, and feedback to promote habit formation [64]. Each action—logging a reading, updating data—yields immediate gratification, which reinforces routine and encourages self-monitoring [65]. Unlike deep narrative or open-ended play, this game is narrowly focused on behavioral reinforcement, positioning it effectively within a reward-centric framework while maintaining potential for future layering of meaning.

#### 5.2.5 Exercise Game:

The exercise game embraces playification [65], framing bodily activity not just as goal-directed but as an opportunity for creative expression and emergent fun. Echoing bodystorming [65] and play-centric design [66], the design leaves space for players to interpret movement tasks in playful and personally meaningful ways. It reflects a departure from gamified exercise trackers toward deep games—those that prioritize emotional resonance and personal interpretation [67]. The game also gestures toward Play Matters [68], highlighting how playful expression can emerge even within structured health interventions. While this module is less visually rigid than others, its strength lies in its invitation to engage the body as both subject and interface for playful exploration, in line with Superbetter [69], where small, embodied actions build toward sustained personal change.

### 5.3 Future work

The next step in our research agenda is to conduct play-testing sessions using the medium-fidelity prototypes developed. These sessions will be crucial in evaluating the usability, engagement, and cultural resonance of each app. To structure this phase, we plan to adopt RITE (Rapid Iterative Testing and Evaluation) methodologies, which allow us to quickly identify usability issues, gather qualitative feedback, and iterate the designs in real-time. In addition to usability testing, the games themselves will be deployed as cultural probes. Participants will engage with the apps not solely as end-users, but as co-researchers exploring and reflecting on their own health practices, cultural narratives, and play preferences. As part of this protocol, we will also collect annotated screenshots and audio commentary, allowing for richer insights into how the games are interpreted and reappropriated in context. This dual function—serving both as playful interventions and as open-ended probes—positions the games as dynamic tools for situated inquiry, extending the participatory ethos of the workshop into future stages of design research. While the current prototypes are modular, each focused on a single hypertension management factor, this structure reflects the design preferences expressed by participants during the workshop. The apps will eventually be hosted on a unified platform called CardioCare Quest, which will organize the five games by factor so users can engage with them based on their own needs and interests.

### 5.4 Limitations

While this study produced rich insights and promising design artifacts, there are several limitations to acknowledge. First, the sample size was relatively small and, while participants represented diverse perspectives within their community, the findings cannot be readily generalized to all Indigenous populations. Second, this study focused on medium-fidelity prototyping, which limits our ability to assess long-term engagement or real-world behavior change. Participants interacted with wireframes and concept sketches rather than fully functional applications, which may not accurately reflect how they would use or respond to the final products. The final notable limitation of this work is the absence of empirical validation, while the prototypes and speculative games were developed through participatory workshop and grounded in relevant theoretical frameworks, they have not yet been tested with users in long-term or real-world settings. As such, claims about their effectiveness, engagement, or cultural resonance remain provisional. This paper prioritizes design exploration and intermediate-level knowledge over evaluation; however, future work will include larger sample sizes, higher fidelity prototyping, and empirical studies that investigate how these games are taken up in practice, how players interpret and interact with the mechanics, and what situated forms of meaning and care emerge through their use.

## 6 Conclusion

This study demonstrates the potential of participatory design and Research through Design methodologies to generate culturally grounded, playful health technologies that support hypertension management in Indigenous communities. By co-creating five medium-fidelity mobile game prototypes with Indigenous participants, we surfaced distinct design particulars tied to culturally meaningful practices, narratives, and ways of knowing. These artifacts not only reflect the lived experiences of participants but also offer playful, accessible strategies for supporting key dimensions of hypertension care. Through the development of an annotated portfolio, we abstracted intermediate design knowledge that can inform future health technology development across contexts. Our findings highlight the value of designing with, rather than for, Indigenous communities, and underscore the importance of culturally responsive approaches in digital health. This work contributes design artifacts, transferable patterns, and methodological insights that together advance inclusive practices in HCI and lay the foundation for future interventions that are culturally resonant, health-focused, and community-led.

## CCS Concepts

• **Human-centered computing** → Human computer interaction (HCI); Human computer interaction (HCI); Interactive systems and tools; Human computer interaction (HCI); HCI design and evaluation methods; • **Social and professional topics** → User characteristics; People with disabilities; User characteristics; Cultural characteristics.
